# Psoriasis: A global perspective

**DOI:** 10.1016/j.jdin.2024.10.002

**Published:** 2024-10-16

**Authors:** Jonathan Kantor

**Affiliations:** Department of Dermatology, Center for Global Health, and Center for Clinical Epidemiology and Biostatistics, Perelman School of Medicine at the University of Pennsylvania, Philadelphia, Pennsylvania; Florida Center for Dermatology, St Augustine, Florida

**Keywords:** burden of disease, epidemiology, global health, psoriasis

*To the Editor:* I am excited to present this virtual special issue dedicated to psoriasis. When I was in training just 2 decades ago, biologic therapy for psoriasis was in its infancy, and yet there was a sense in the air that it was only a matter of time before the widening range of increasingly targeted options would make psoriasis management an easy—if perhaps increasingly costly—endeavor. While this has partly come to pass, as highlighted in this issue, significant challenges remain.

The range of manuscripts in this issue is truly global. For example, a randomized controlled trial from Thailand suggested a potential role for botulinum toxin in nail psoriasis management,^1^ while another manuscript addressed the impact of disease severity on quality of life in the Philippines.^2^ Still other studies evaluated the effectiveness of risankizumab in a Japanese cohort,^3^ while another explored contact sensitization among psoriasis patients in Lithuania.^4^ Yet another study assessed real-world outcomes in patients with underlying malignancy treated with guselkumab in Spain.^5^

Psoriasis remains a significant challenge, and exploring everything from its epidemiology to associations and therapeutic options remains at the forefront of dermatology. I am excited that this issue contains such a range of submissions, both geographically and topically, and I look forward to the continued productivity of my colleagues around the world as they continue to push the envelope on what is possible. Ultimately, this relentless drive to present a range of therapeutic options will help to hopefully make good on the promise of decades ago so that no psoriasis patient will be left behind.

## Conflicts of interest

None disclosed.
